# Production of coffee-dyed bacterial cellulose as a bio-leather and using it as a dye adsorbent

**DOI:** 10.1371/journal.pone.0265743

**Published:** 2022-03-24

**Authors:** Hyunjin Kim, Hye Rim Kim

**Affiliations:** Department of Clothing and Textiles, Sookmyung Women’s University, Seoul, South Korea; King Saud University, SAUDI ARABIA

## Abstract

Owing to its sustainability and environmentally friendliness, bacterial cellulose (BC) has received attention as a zero-waste textile material. Since the color of original BC was mostly yellowish white, a dyeing process is necessary to suggest BC as a textile. Thus, this study aimed to suggest a natural dyeing method using coffee to produce an eco-friendly coffee-dyed bacterial cellulose (BC-COF) bio-leather and to propose a reusing method as a dye adsorbent. To determine the dyeing and mordanting conditions with the highest color strength value, parameters such as dyeing temperature, time, mordanting methods were evaluated. Fourier-transform infrared spectroscopy and X-ray diffraction analysis confirmed that BC-COF was successfully colorized with coffee without changing its chemical and crystalline structures. In addition, field-emission scanning electron microscopy and Brunauer-Emmett-Teller surface area analysis confirmed that coffee molecules were successfully incorporated into fiber structures of BC. The effects of pH, concentration, temperature, and time on the adsorption of methylene blue dye using BC-COF bio-leather were also evaluated using ultraviolet-visible spectroscopy and zeta potential measurement. The results showed that BC-COF was found to be most effective when pH 6 of methylene blue solution with a concentration of 50 mg/L was adsorbed for 30 minutes at 25°C. Moreover, BC-COF could be reused for multiple times and had better dye adsorption rate compared to the original BC. From the results, it was confirmed that BC-COF could be employed as a dye adsorbent.

## Introduction

With the demand for sustainable and eco-friendly fashion increasing significantly, researchers have been focusing on the development of bio-leather as an alternative to the natural leather sourced from animals [1, 2]. Bio-leather is a bio-material derived from natural non-animal materials [2]. By using bio-leather, it can prevent unnecessary slaughter to obtain animal leathers [3]. Moreover, since bio-leather is biodegradable, the amount of textile waste can be reduced after use [1]. Owing to its sustainable and environment-friendly properties, bio-leather is gaining attention as a substitute to animal leather. Currently known sources for bio-leather include pineapple, mushroom, jellyfish, cork, and bacterial cellulose (BC) [2, 4, 5]. Among these various types of bio-leather, we have focused on BC.

BC is a pure cellulose bio-material produced by the synthesis of bacteria such as *Acetobacter xylinus* and *Komagataeibacter* [6]. It looks like a gelatinous pellicle with good resiliency [1], and composed of three-dimensional interconnected nanofibers [7], leading to high porosity, good water-holding capacity, and high crystallinity [8]. BC is cost-effective owing to its shortened production process that when BC pellicle is in a dried state, it resembles the leather [2, 9]. Moreover, BC bio-leather is eco-friendly and non-toxic with excellent biodegradability and biocompatibility [10]. Furthermore, the excellent moldability and the leather-like appearance have expanded the application of BC as a zero-waste sustainable textile material [2, 11].

Since the original color of BC is mostly transparent or yellowish white, the dyeing process is essential for introducing various colors to BC bio-leather [12]. Natural dyes are non-toxic, non-carcinogenic, biodegradable, and do not cause environmental pollution as they do not generate toxic wastewater [13]. Moreover, natural dyes have delicate colors compared to synthetic dyes, and possess antibacterial and antioxidant functionalities that are absent in synthetic dyes [14]. Considering these advantages, natural dyes are expected to maximize the eco-friendly aspect of BC bio-leather. Among the various natural dyes, coffee was selected for this study. Coffee is a tannin-based brown dye that can be easily obtained in everyday life [15]. It is mainly composed of tannin, protein, caffeine, ash, and sugar [16]. Due to the increasing coffee consumption, the amount of spent coffee grounds, which is a byproduct of coffee consumption, is also increasing [17]. Hence, it is highly economical to reuse spent coffee grounds as natural dyes [15, 18]. Nevertheless, despite the advantages of natural dyeing, there are limited studies on the natural dyeing of BC bio-leather [4, 19, 20].

Taking the sustainability factor into consideration, we have focused on the idea of using the colored BC bio-leather for water treatment. Nowadays, with the development of industry, water pollution by various pollutants is considered as a serious problem. In particular, synthetic dyes such as methylene blue [21, 22], Amido Black [23], and Prussian Blue [24] used in the textile dyeing industry, pesticides such as fenuron used in the agricultural industry [25], heavy metal ions including cadmium, nickel, copper, lead, and cobalt [26] are widely known sources of water pollution. To remove these pollutants from water, previous studies on various water treatment methods including adsorption, biosorption, catalysis, photodegradation, electrochemical process, ultrafiltration, reverse osmosis, coagulation, magnetic separation, and oil cake treatment have been reported [22, 23, 27–31]. Among the methods mentioned, adsorption is the most widely used method due to its high efficiency, cost-effectiveness, and simple process [26, 28, 32]. Several studies have used BC as an adsorbent for heavy metals [33, 34] and dyes [35–37] in wastewater. However, BC used as an adsorbent in those studies was applied with synthetic polymers instead of natural substances and was not used for fabric purposes. Thus, this study proposes a natural dyeing method for BC as a bio-leather and the reuse method as a dye adsorbent after use.

Therefore, this study aims to recommend a natural dyeing method to produce an eco-friendly colored BC bio-leather, and to propose a reusing method for colored BC bio-leather to purify dyeing wastewater. This is the first study to apply coffee to BC as a dyestuff and examine the reusability of colored BC bio-leather. Here in, the coffee-dyed BC bio-leather is referred to as BC-COF. The dyeing conditions of BC-COF, such as the extraction of coffee, dyeing time, and dyeing temperature, were determined based on the color strength (*K/S*) measurement results. The mordanting condition of BC-COF was selected based on the percent change in the residual color strength after the washing durability test. The physical and chemical structures of BC-COF were examined by Fourier-transform infrared spectroscopy (FT-IR), X-ray diffraction (XRD), field-emission scanning electron microscopy (FE-SEM), and Brunauer-Emmett-Teller (BET) surface area analysis. To evaluate the adsorption performance of BC-COF bio-leather, methylene blue dye was used as a simulated water pollutant. The reusability of BC-COF as a dye adsorbent was observed by varying the concentration of the aqueous solution, adsorption time, adsorption temperature, pH level of the aqueous solution, and the number of repeated adsorptions.

## Materials and methods

### Materials

Kombucha SCOBY (Symbiotic Culture of Bacteria and Yeast) was obtained from GetKombucha (CO, USA). Peptone and yeast extract were purchased from BD Biosciences (CA, USA). Glucose (98.0%), hydrogen peroxide (H_2_O_2_, 34.5%), sodium hydroxide (NaOH, 98%), acetic acid (99.0%), potassium chloride (KCl, 99%), and methylene blue (C. I. 52015, 95.0%) were sourced from Duksan Pure Chemical Co., Ltd. (Seoul, Korea). 0.1 N hydrogen chloride (HCl) solution and 0.1 N NaOH solution were purchased from Samchun Chemical Co., Ltd. (Seoul, Korea). Ground coffee powder was obtained from LION Coffee (HI, USA). The mordants selected for this study were aluminum potassium sulfate (AlK(SO_4_)_2_∙12H_2_O, 99.2%), copper (II) sulfate (CuSO_4_∙5H_2_O, 99.0%), and iron (II) sulfate (FeSO_4_∙7H_2_O, 98.0~102.0%), which were purchased from Duksan Pure Chemical Co., Ltd. (Seoul, Korea). All chemical reagents were used as received without additional purification.

### Cultivation and pre-treatment of BC

BC pellicle was cultured based on a method by Han et al. [38] and was undergone pre-treatment processes such as washing, bleaching, and swelling [38, 39]. In brief, a carbon source (glucose; 20 g/L) and a nitrogen source (peptone and yeast extract mixture; 5 g/L for each) were combined thoroughly with distilled water to produce the Hestrin and Schramm (HS) medium. Kombucha Symbiotic Culture of Bacteria and Yeast (SCOBY) was placed in the HS medium in a fabric-to-liquor ratio of 1:7 (w/v) and cultured statically at 26°C for eight days to obtain a 1 cm-thick BC. Thereafter, the BC was washed with a 3% NaOH solution at 25°C for 90 minutes with shaking at 50 rpm in a shaking water bath (BS-31; JEIO TECH Co., Daejeon, Korea), and neutralized with an acetic acid solution (pH 3.0) at 25°C for 30 minutes with shaking at 50 rpm. Subsequently, the BC was bleached with a 5% H_2_O_2_ solution at 90°C for 60 minutes with shaking at 110 rpm, swelled with an 8% NaOH solution in a fabric-to-liquor ratio of 1:10 (w/v) for 30 minutes in an ultrasonic bath (UC-20; JEIO TECH Co., Daejeon, Korea), and neutralized with an acetic acid solution (pH 3.0) at 50°C for 30 minutes.

### Extraction of coffee

The extraction condition of coffee dye was referred to the method of Song and Kim [40] with some modifications. Before the coloration, coffee at a concentration of 200 weight percent (wt%) of BC was added to distilled water in various extraction liquor ratios of 1:10, 1:20, 1:50, and 1:100 (w/v) and extracted at 90°C for 1 h. The extracted coffee solution was then cooled down to room temperature and filtered to eliminate insoluble residues.

### Production of BC-COF bio-leather

The pre-treated BC was dyed with coffee to produce BC-COF bio-leather. Thereafter, the pre-treated BC with a size of 3 cm x 3 cm was immersed in the coffee solution with a fabric-to-liquor ratio of 1:10 (w/v) with shaking at 80 rpm in a shaking water bath. To determine the dyeing condition of BC-COF, varied dyeing temperatures ranging from 40°C to 80°C and varied dyeing time ranging from 30 minutes to 5 h were evaluated. After dyeing, BC-COF was washed thoroughly with distilled water and dried at 50°C for 2 h.

### Mordanting of BC-COF bio-leather

BC-COF was mordanted by pre-, meta-, and post-mordanting methods using the aforementioned mordants with 3% (o.w.f.) aluminum potassium sulfate (AlK(SO_4_)_2_), 2% (o.w.f.) copper (II) sulfate (CuSO_4_), and 1% (o.w.f.) iron (II) sulfate (FeSO_4_), respectively.

For the pre-mordanting method, pre-treated BC was immersed in a mordanting solution in a fabric-to-liquor ratio of 1:10 (w/v) at 40°C for 20 minutes with shaking at 80 rpm. Subsequently, pre-mordanted BC was dyed in the extracted coffee solution with the determined dyeing conditions.

For the meta-mordanting method, mordants were added directly to the extracted coffee solution and dissolved thoroughly, and mordanted with the determined dyeing conditions of BC-COF.

For the post-mordanting method, BC-COF was placed in a mordanting solution in a fabric-to-liquor ratio of 1:10 (w/v) at 40°C for 20 minutes with shaking at 80 rpm. After the dyeing process was completed, the BC-COF was dried in a drying oven (OF-22G; JEIO TECH Co., Daejeon, Korea) for 24 h at 25°C.

### Characterizations of BC-COF bio-leather

The dyeing conditions of BC-COF, such as extraction of coffee, dyeing time, and dyeing temperature, were determined by the color strength (*K/S*) obtained using a spectrophotometer (CM-2600d; Konica Minolta Sensing, Inc., Tokyo, Japan) under a standard illuminant D_65_ at 10° observer settings. The color values with the CIELAB color system were expressed using L*, a*, and b* coordinates, where L* value is a measure of lightness, ranging from 0 (black) to 100 (white); positives and negatives in a* value represent redness and greenness, and positives and negatives in b* value represent yellowness and blueness. The color strength (*K/S*) of the samples was calculated according to the Kubelka-Munk Eq (1):

$$K/S=\frac{{\left(1‐R\right)}^{2}}{2R}$$ (1)

where *K* is the absorbance coefficient, *S* is the scattering coefficient, and *R* is the reflectance ratio of the sample. All measurements were performed five times for each sample, and the average value was presented.

The mordanting condition of BC-COF bio-leather was evaluated according to ISO 105-E01:2013 with some modifications. Washing durability tests were carried out with three sequential washing steps. Each step was held with a fabric-to-liquor ratio of 1:200 (w/v) at 25°C for 30 minutes with shaking at 110 rpm. After each step, the mordanted BC-COF samples were dried at 25°C for 24 h. The *K/S* values of the samples were measured after each washing step, and the residual color strength was calculated using Eq (2):

$$\mathrm{Residual}\;\mathrm{color}\;\mathrm{strength}\;(\%)=100‐\left|\frac{W_{A}‐W_{B}}{W_{B}}\right|\times 100$$ (2)

where *W*_*A*_ stands for the *K/S* value after washing, and *W*_*B*_ stands for the *K/S* value before washing.

FT-IR (Nicolet IS50; Thermo Fisher Scientific, Waltham, MA, USA) was used to examine the chemical structure of the BC-COF. The FT-IR spectra of samples were measured in the wavenumber range of 800–4,000 cm^-1^ at a resolution of 0.4 cm^-1^ with 32 scans. Baseline normalization was performed for each spectrum using the OMNIC software (Thermo Fisher Scientific, Waltham, MA, USA).

The crystalline structure of BC-COF was analyzed using a D8 ADVANCE diffractometer (Bruker AXS Inc., Fitchburg, WI, USA). XRD measurements were carried out with a Cu-Kα radiation source (λ = 1.5406 nm) at 40 kV and 40 mA in the 2θ range of 0–40° with a measurement speed of 0.1°/min. The degree of crystallinity (%) was calculated according to Segal et al. [41] (Eq (3)):

$$\mathrm{The}\;\mathrm{degree}\;\mathrm{of}\;\mathrm{crystallinity}\;(\%)=\left|\frac{I_{o}‐I_{a}}{I_{o}}\right|$$ (3)

where *I*_*o*_ stands for the height of the total intensity and *I*_*a*_ stands for the heights of the amorphous region of the XRD diffraction pattern.

The surface morphology of the BC-COF was characterized by FE-SEM (JSM-7600F; JEOL Ltd., Tokyo, Japan). All samples were platinum-coated using a magnetron sputter coater (108 auto; Cressington Scientific Instruments, Watford, UK) before observation, and then observed at an accelerated voltage of 3 kV.

The specific surface area was measured according to the Brunauer-Emmett-Teller (BET) method, and the Barrer-Joyner-Halenda (BJH) model was used to calculate the pore size distribution. The BET surface area and pore size were examined through N_2_ adsorption/desorption experiments at 77 K using the Surface Area and Pore Size Analyzer (QUADRASORB evo™, Quantachrome Corporation, Boynton Beach, FL, USA). The samples were placed in sample cells and heated up to 120°C for 24 h to remove the moisture and then cooled down to room temperature before the analysis.

### Evaluation of the reusability of BC-COF bio-leather as a dye adsorbent

BC-COF was placed in the methylene blue aqueous solution at various concentrations (20, 50, 100 mg/L) in a fabric-to-liquor ratio of 1:200 (w/v) and adsorbed at a fixed temperature of 25°C for various adsorption times with shaking at 80 rpm. The dye adsorption properties of BC-COF were evaluated by varying the concentration and pH level of the aqueous solution, adsorption time, adsorption temperature, and number of repeated adsorptions by measuring the ultraviolet-visible (UV-Vis) spectra of residual aqueous solution. The spectra of the samples were recorded using a UV-Vis spectrophotometer (SynergyMx; Shimadzu, Kyoto, Japan) in the wavelength range of 300–800 nm. The maximum wavelength for methylene blue was 660 nm. All measurements were performed five times, and the average value was presented.

The dye adsorption rate of BC-COF was calculated according to Eq (4):

$$\mathrm{Dye}\;\mathrm{adsorption}\;\mathrm{rate}\;(\%)=\left|\frac{U_{A}‐U_{B}}{U_{B}}\right|$$ (4)

where *U*_*A*_ stands for the absorbance of the residual aqueous solution at 660 nm after the adsorption, and *U*_*B*_ is the absorbance of the aqueous solution at 660 nm before the adsorption.

The zeta potential value of BC-COF bio-leather was measured by using Nanoparticle Analyzer (SZ-100; Horiba Ltd., Kyoto, Japan). 6 mm carbon electrode cell was utilized at 25°C, and Smoluchowski’s approximation was used to convert the electrophoretic mobility into zeta potential value [42]. The measurements were performed in a 0.01 N KCl solution, and the pH values were adjusted with 0.1 N HCl or 0.1 N NaOH solutions. All measurements were performed five times, and the average value was presented.

## Results and discussion

### Dyeing conditions of BC-COF bio-leather

The dyeing conditions of BC-COF bio-leather were investigated by the *K/S* value since it is directly proportional to the amount of color present on the surface of BC bio-leather [43]. The dyeing conditions were as follows: extraction of coffee, dyeing time, dyeing temperature, and mordanting conditions.

**Fig 1:**
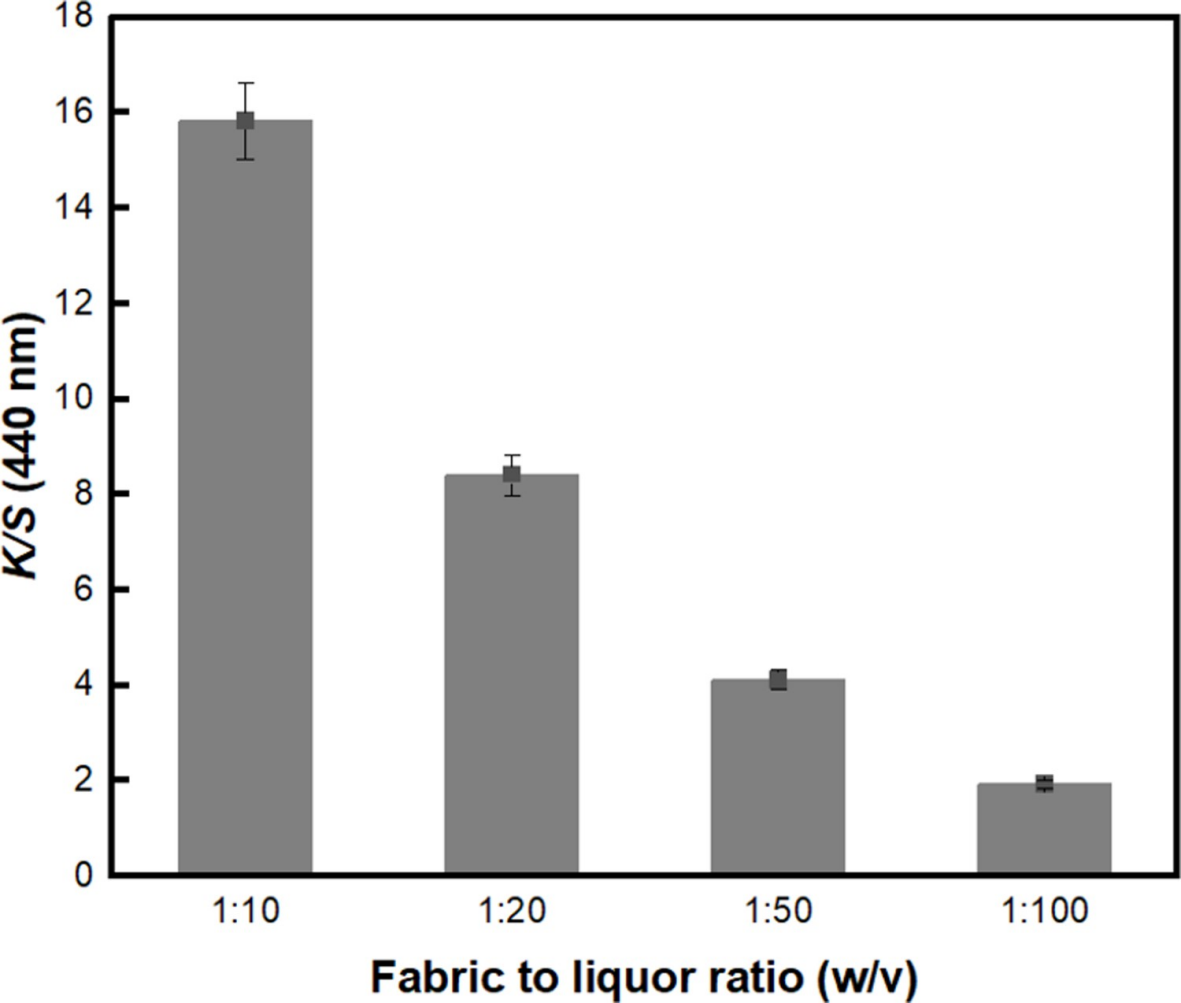
The *K/S* values of BC-COF bio-leather at 440 nm with varied extraction liquor ratios (Extraction conditions: Coffee at an amount of 200 wt% BC, 90°C, 1 h; dyeing conditions: Fabric-to-liquor ratio of 1:10 (w/v), 70°C, 30 min).

First, the extraction conditions for coffee were examined. The amount of coffee was fixed at 2 times of the weight of BC, and the extraction liquor ratios varied from 1:10 to 1:100 (w/v). As shown in Fig 1, the highest *K/S* value was observed in the sample with an extraction liquor ratio of 1:10 (w/v). This finding is in accordance with the extraction conditions reported in a previous study [44]. Unlike other kinds of natural dyes [45, 46], coffee might need to be extracted at a high concentration to obtain a deep color after dyeing. Thus, a liquor ratio of 1:10 (w/v) was selected as the extraction condition for producing BC-COF bio-leather.

**Fig 2:**
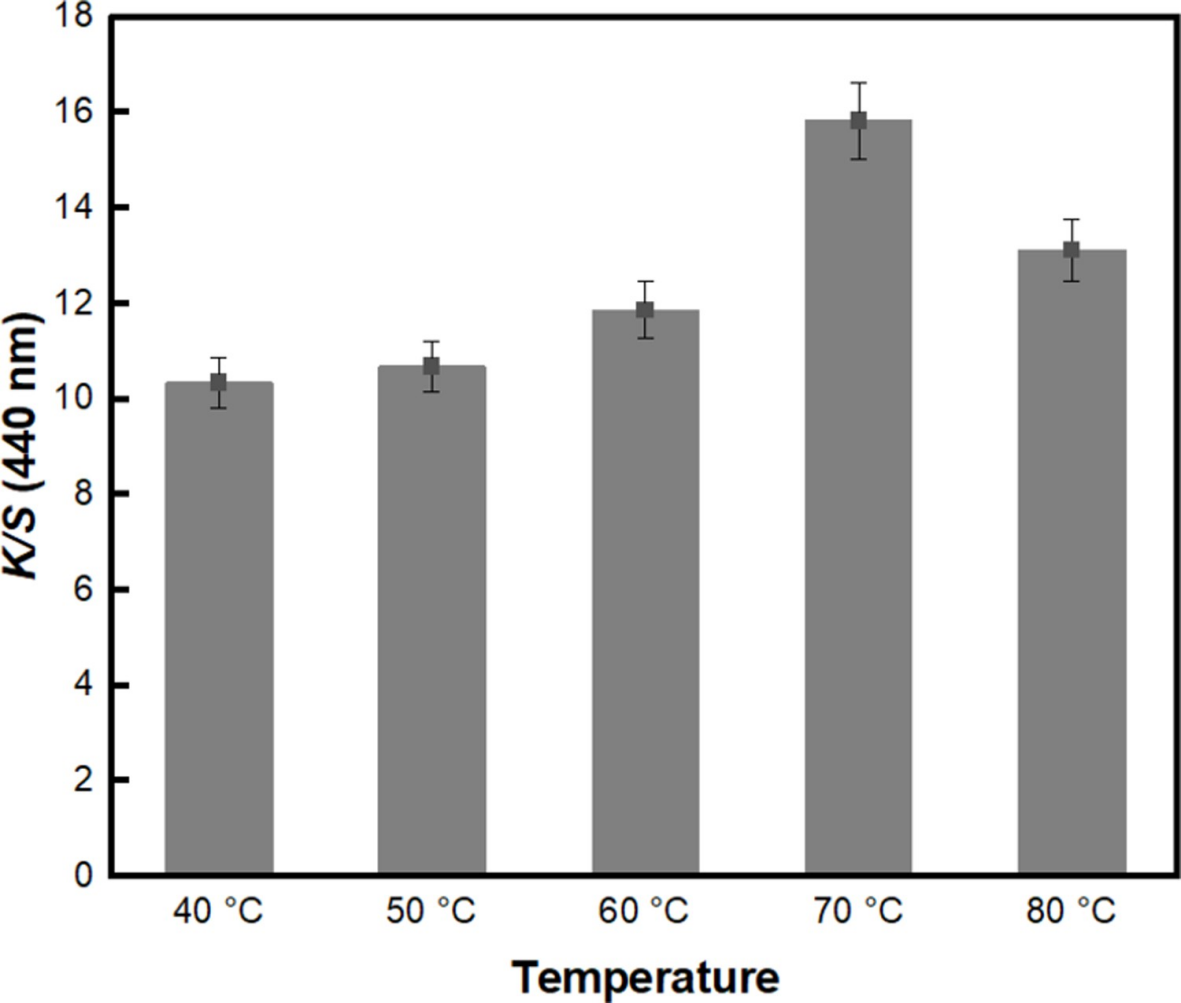
The *K/S* values of BC-COF bio-leather at 440 nm with varied dyeing temperature from 40°C to 80°C (Extraction conditions: Liquor ratio of 1:10 (w/v), coffee at an amount of 200 wt% BC, 90°C, 1 h; dyeing conditions: Fabric-to-liquor ratio of 1:10 (w/v), 30 min).

Subsequently, the dyeing temperature of BC-COF bio-leather was observed by varying the temperature from 40°C to 80°C. Fig 2 demonstrates that the *K/S* value of BC-COF gradually increased as the dyeing temperature rose and reached the highest value at 70°C. This is the usual effect of temperature on the increase of the dyeing rate. Most dye molecules exist as aggregates at lower temperature although only individual dye molecules could penetrate into the fibers [47]. Hence, as the temperatures increases, the aggregates gradually break up so that more individual molecules are able to penetrate into the fibers [47].

However, as the temperature exceeded 70°C, the *K/S* value of the BC-COF bio-leather decreased. This could be related to the state of dye molecules in the dyeing solution. As the dyeing temperature increases further, there would be no aggregates left to break up, leading to the decreased dyeability with further increased temperature [47]. Thus, 70°C was selected as the dyeing temperature for the BC-COF bio-leather.

**Fig 3:**
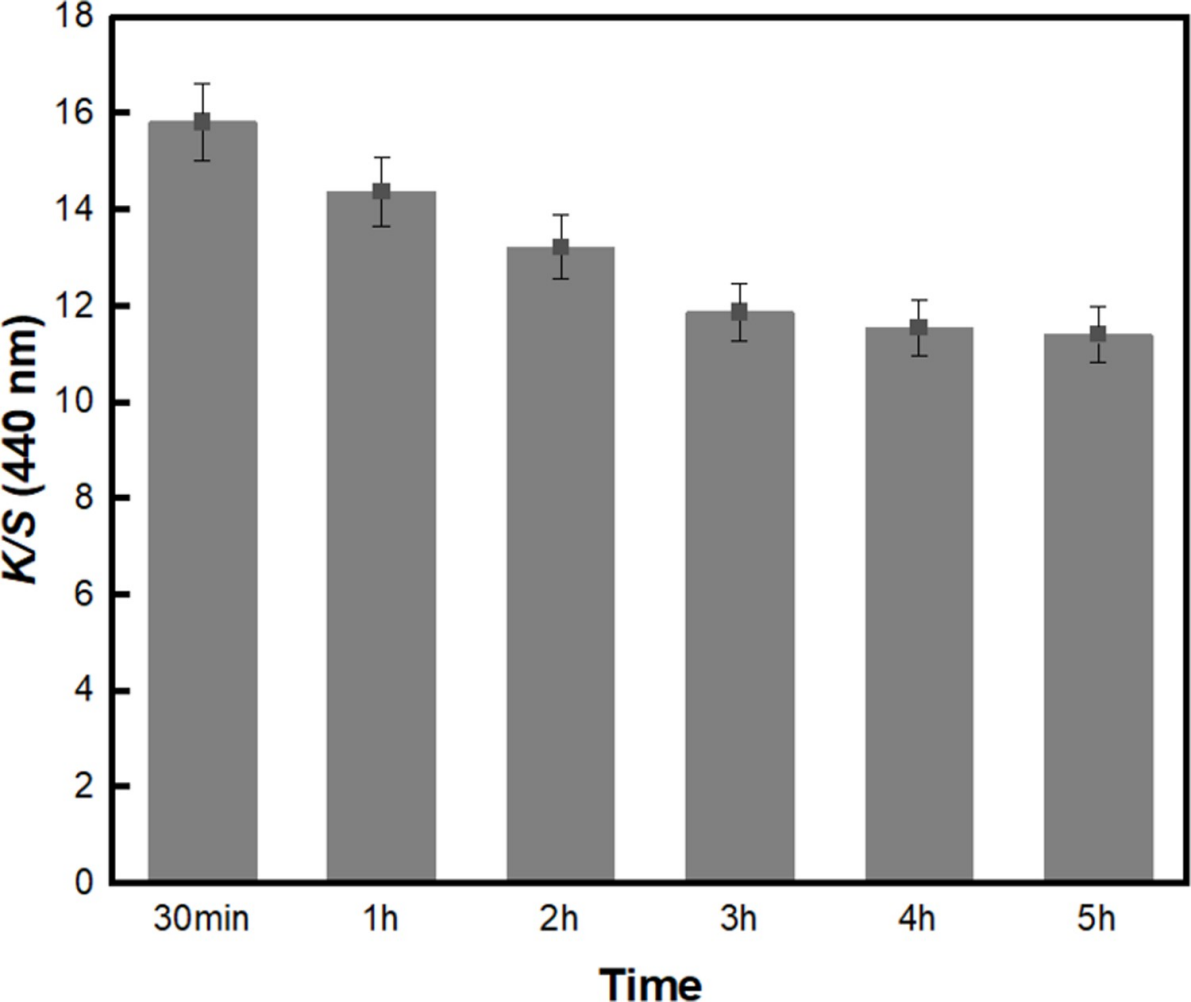
The *K/S* values of BC-COF bio-leather at 440 nm with varied dyeing time from 30 min to 5 h (Extraction conditions: Liquor ratio of 1:10 (w/v), coffee at an amount of 200 wt% BC, 90°C, 1 h; dyeing conditions: Fabric-to-liquor ratio of 1:10 (w/v), 70°C).

Lastly, the dyeing time of BC-COF bio-leather was evaluated by varying the time from 30 minutes to 5 h. As demonstrated in Fig 3, the *K/S* value of BC-COF bio-leather gradually decreased as the dyeing time increased. This can be attributed to the limited number of coffee pigment molecules that can penetrate the BC fiber structure. Consequently, similar to the temperature condition, only few aggregated dye molecules were left to break up and diffuse into the fibrous structure of BC as the dyeing time increases [47]. Thus, the dyeing time of BC-COF bio-leather was determined to be 30 minutes.

### Mordanting condition of BC-COF bio-leather

**Fig 4:**
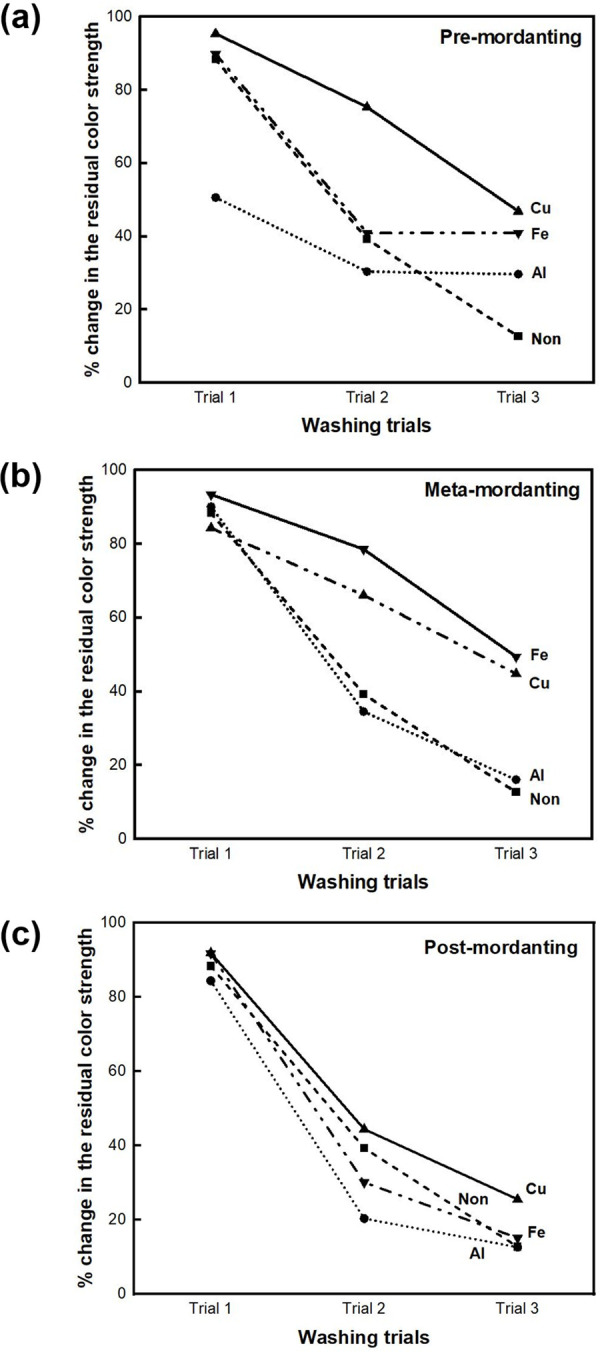
The percent change in the residual color strength of BC-COF bio-leather (a) pre-mordanted with AlK(SO_4_)_2_, CuSO_4_, and FeSO_4_; (b) meta-mordanted with AlK(SO_4_)_2_, CuSO_4_, and FeSO_4_; (c) post-mordanted with AlK(SO_4_)_2_, CuSO_4_, and FeSO_4_ (Extraction conditions: liquor ratio of 1:10 (w/v), coffee at an amount of 200 wt% BC, 90°C, 1 h; Dyeing conditions: fabric-to-liquor ratio of 1:10 (w/v), 70°C, 30 min).

The mordanting condition of the BC-COF bio-leather was determined by the percent change in the residual color strength after the washing durability test. As indicated in Fig 4, after three sequential washing steps, it was confirmed that the washing durability of all the mordanted BC-COF samples was better than that of the non-mordanted BC-COF sample. This can be attributed to the low affinity of coffee pigment with BC fibers [44], thereby mordanting is essential for improving the washing durability of the BC-COF. Among all the mordanted BC-COF samples, a sample meta-mordanted with FeSO_4_ maintained approximately 49.4% of its original color after three repeated washing trials. Thus, the mordanting condition for BC-COF bio-leather was determined to be meta-mordanting with 1% FeSO_4_.

### Surface characterizations of BC-COF bio-leather

The surface characterizations of BC-COF bio-leather were performed using FT-IR, XRD, FE-SEM, and BET. The original BC, which had undergone washing, bleaching, and swelling pre-treatments, was selected as the control sample.

**Fig 5:**
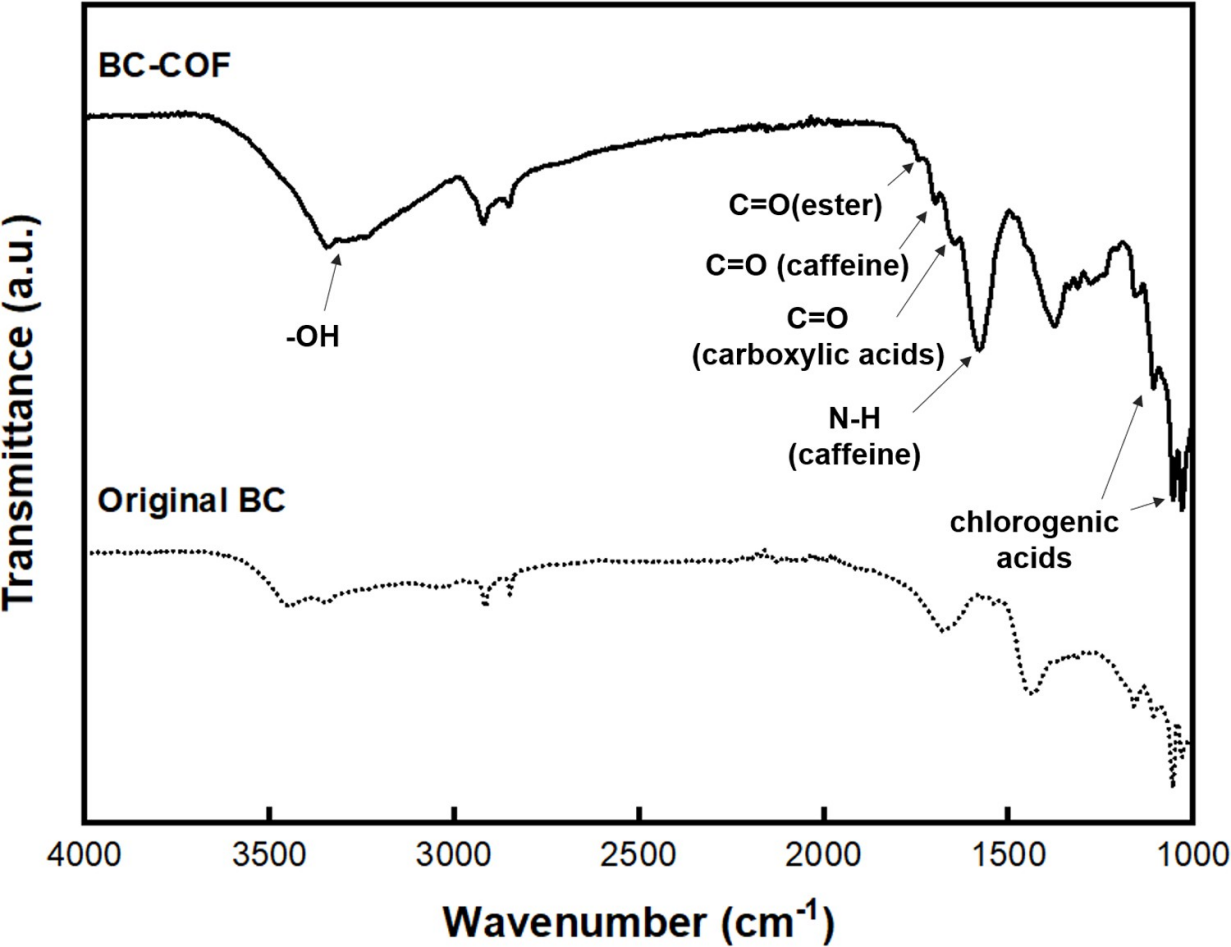
FT-IR spectra of original BC and BC-COF bio-leather (Extraction conditions: Liquor ratio of 1:10 (w/v), coffee at an amount of 200 wt% BC, 90°C, 1 h; dyeing conditions: Fabric-to-liquor ratio of 1:10 (w/v), 70°C, 30 min, meta-mordanting with 1% of FeSO_4_).

FT-IR was used to investigate the chemical structure of BC-COF bio-leather. The FT-IR spectra are demonstrated in Fig 5, and the characteristics of the BC-COF peaks are listed in Table 1. As shown in Fig 5, the FT-IR spectra of BC-COF and original BC displayed peaks at 3347 cm^-1^ and 3448 cm^-1^, respectively, which were attributed to the ‒OH stretching vibrations of the hydroxyl groups of cellulose and coffee [48–50]. These peaks indicated that the chemical structure of BC remained unchanged after dyeing with coffee [2]. Also, it was confirmed that the ‒OH stretching vibrations of BC-COF was stronger than that of the original BC due to the hydrogen bonds formed between coffee molecules and fibrous structure of BC during the dyeing process [49]. After dyeing with coffee, some characteristic peaks of coffee were observed in the spectra of BC-COF. First, a peak was observed at 1744 cm^-1^, which was attributed to the C = O stretching vibration of the ester group [51]. The peaks at 1699 cm^-1^ and 1645 cm^-1^ were ascribed to the C = O stretching vibration of aliphatic acids from caffeine [52, 53] and the C = O stretching vibration of carboxylic acids [54], respectively. In addition, the characteristic peaks of coffee at 1650 cm^-1^–1550 cm^-1^ were dependent on the type of coffee beans [55]. In this study, Hawaiian Kona coffee was used to dye BC to produce BC-COF bio-leather, and the peaks were observed at 1580 cm^-1^, indicating the N‒H stretching vibration of caffeine [56]. Lastly, the peaks appearing at 1113cm^-1^ and 1053 cm^-1^ could also be associated with chlorogenic acids [57, 58], which are a large family of esters formed by quinic acid and certain trans-cinnamic acids [59]. Thus, the FT-IR analysis confirmed that BC-COF was successfully dyed with coffee, and the cellulosic peak of BC was still observed after dyeing with coffee.

**Table 1:** Characteristics of peaks of original BC and BC-COF bio-leather (Extraction conditions: Liquor ratio of 1:10 (w/v), coffee at an amount of 200 wt% BC, 90°C, 1 h; dyeing conditions: Fabric-to-liquor ratio of 1:10 (w/v), 70°C, 30 min, meta-mordanting with 1% of FeSO_4_).

Wavenumber (cm^-1^)			Peak assignment
Peak no.	Original BC	BC-COF	
1	3448	3347	‒OH stretching [48, 50]
2	-	1744, 1699, 1645	C = O stretching [51–54]
3	-	1580	N‒H stretching [56]
4	-	1113, 1053	chlorogenic acids [57, 58]

**Fig 6:**
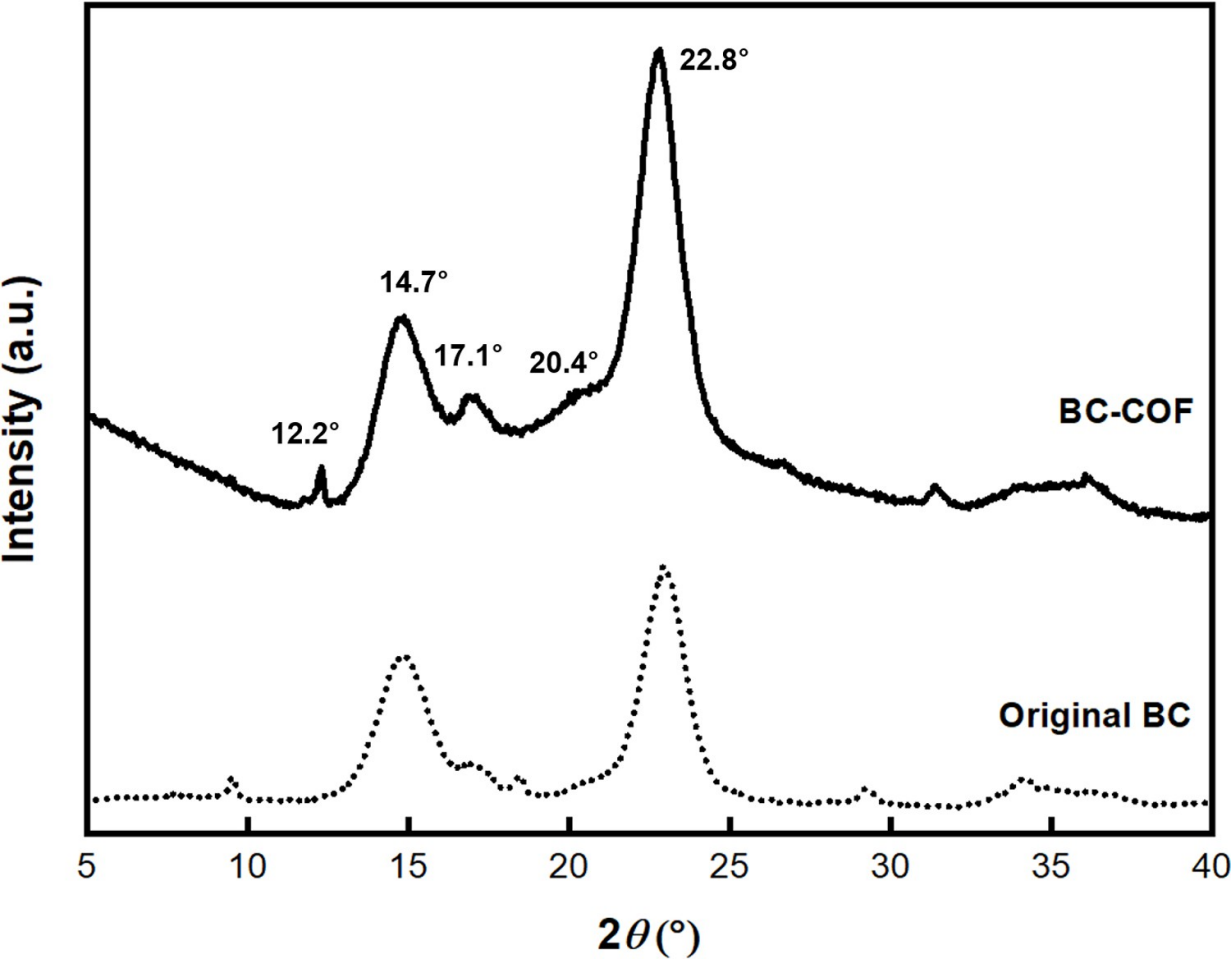
XRD diffraction patterns of original BC and BC-COF bio-leather (Extraction conditions: Liquor ratio of 1:10 (w/v), coffee at an amount of 200 wt% BC, 90°C, 1 h; dyeing conditions: Fabric-to-liquor ratio of 1:10 (w/v), 70°C, 30 min, meta-mordanting with 1% of FeSO_4_).

XRD analysis was performed to examine the crystalline structure of the BC-COF bio-leather. In Fig 6, three peaks at 2*θ* = 14.7°, 17.1°, and 22.8° were observed in the diffraction patterns of both the original BC and BC-COF. These peaks were ascribed to the cellulose I peaks of BC [60, 61], indicating that BC-COF maintained its crystalline structure after natural dyeing with coffee. The change in the crystalline structure might affect the mechanical properties of BC such as tensile strength and thermal stability [62]. Thus, maintaining the crystalline structure of BC is important. This finding is in accordance with the FT-IR analysis, which has been previously discussed. In the diffraction pattern of BC-COF, a sharp peak at 2*θ* = 12.2° and an amorphous peak at 2*θ* = 20.4° were observed. These two peaks correspond to the characteristic peaks of roasted coffee [63], implying that the coffee molecules were entrapped inside the BC fiber structure. In addition, the peaks appeared at 2*θ* = 29.4° and 34.1° were slightly shifted to 31.4° and 36.1°, respectively. This could be explained that the crosslinking between BC fibers and coffee molecules increased the chain-chain distance inside BC, leading to the XRD peak shift after dyeing with coffee [64].

Table 2 describes the degree of crystallinity of BC-COF bio-leather. According to Table 2, the degree of crystallinity of BC decreased slightly after dyeing with coffee. This tendency could be associated with the breakdown of hydrogen bonds in BC due to the crosslinking between BC fibers and coffee [4]. Thus, the XRD results confirmed that BC-COF was successfully dyed with coffee by maintaining its crystalline cellulose structure.

**Table 2:** The degree of crystallinity (%) of original BC and BC-COF bio-leather (Extraction conditions: Liquor ratio of 1:10 (w/v), coffee at an amount of 200 wt% BC, 90°C, 1 h; dyeing conditions: Fabric-to-liquor ratio of 1:10 (w/v), 70°C, 30 min, meta-mordanting with 1% of FeSO_4_).

Sample	The degree of crystallinity (%)
Original BC	82.6 ± 3.6
BC-COF	78.2 ± 6.5

**Fig 7:**
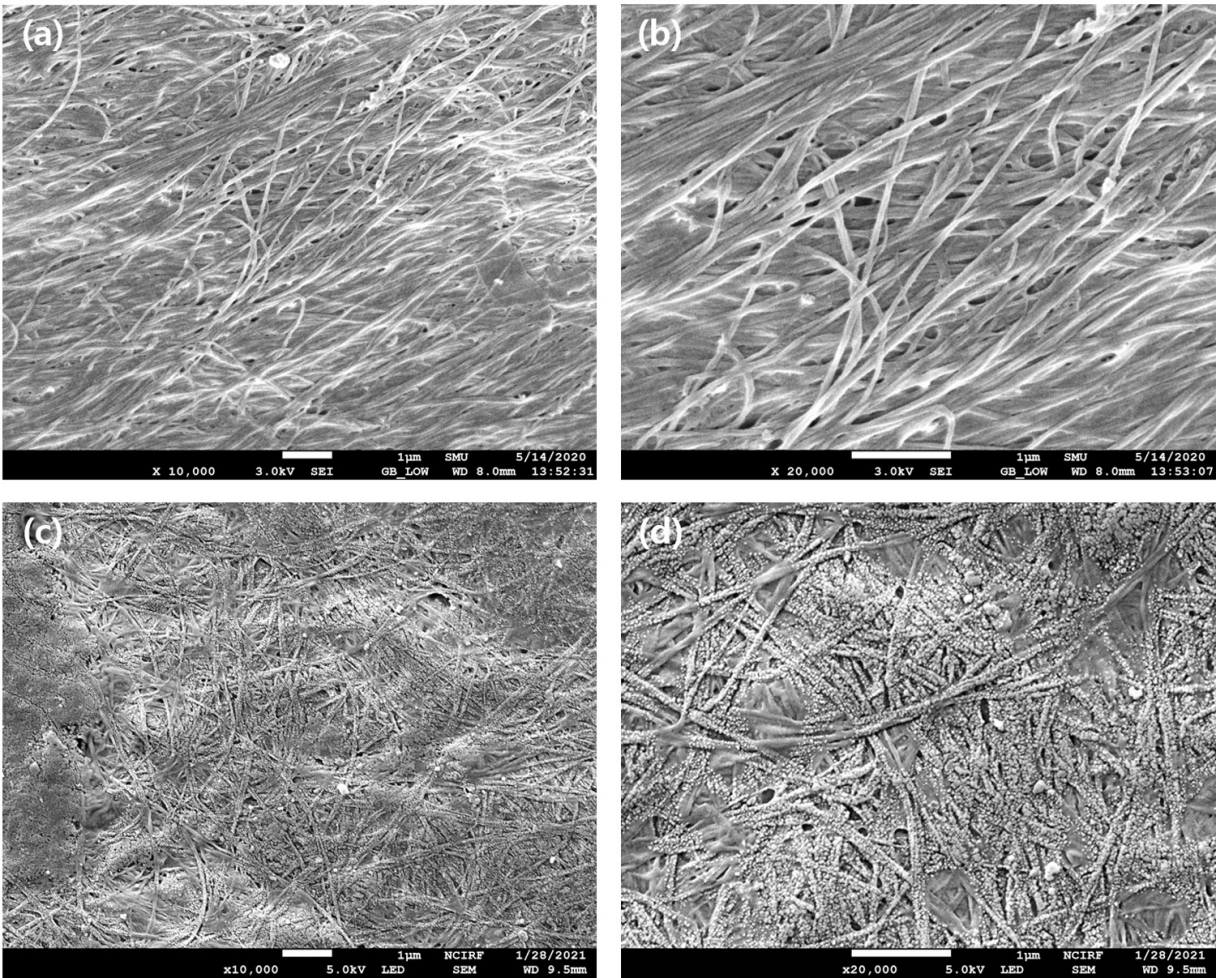
SEM images of **a, b** original BC; **c, d** BC-COF bio-leather at 10,000 and 20,000x magnification, respectively, with a scale bar of 1 μm (Extraction conditions: liquor ratio of 1:10 (w/v), coffee at an amount of 200 wt% BC, 90°C, 1 h; Dyeing conditions: fabric-to-liquor ratio of 1:10 (w/v), 70°C, 30 min, meta-mordanting with 1% of FeSO_4_).

The surface morphology of the BC-COF bio-leather was examined using FE-SEM. In Fig 7A and 7B, the fine cellulose nanostructure is clearly visible in the original BC. Compared to the original BC, different morphologies were observed after coloration with coffee. In the SEM image of BC-COF (Fig 7C and 7D), spherical coffee molecules penetrated the BC nanostructures and attached to the fiber strands. This change in the surface of BC-COF could also be explained by the crosslinking between BC fibers and coffee [4], as observed by XRD. From the SEM analysis results, it was confirmed that the color of BC-COF bio-leather was due to the penetration and deposition of coffee particles inside the BC fiber structures.

The surface area and porous features of BC-COF bio-leather was examined by using the BET method. According to Table 3, both the surface area and pore diameter decreased after coloration with coffee. Since the compact and dense filling of pores leads to the reduction in the surface area and porosity [65], a possible explanation would be that the coffee molecules in the extracted coffee solution penetrated into the porous structure of BC and filled up the pores. The BET result was corresponding to the results from SEM images that BC-COF bio-leather had fewer empty spaces among BC fibrils compared to original BC. Thus, it was confirmed that coffee molecules were incorporated into the BC fiber structures and reduced the surface area and pore size.

**Table 3:** BET surface areas and pore diameters of original BC and BC-COF bio-leather (Extraction conditions: Liquor ratio of 1:10 (w/v), coffee at an amount of 200 wt% BC, 90°C, 1 h; dyeing conditions: Fabric-to-liquor ratio of 1:10 (w/v), 70°C, 30 min, meta-mordanting with 1% of FeSO_4_).

Sample	Surface area (m^2^/g)	Pore diameter (nm)
Original BC	13.5 ± 3.2	3.1 ± 0.5
BC-COF	11.6 ± 1.4	3.0 ± 0.6

### Reusability of BC-COF bio-leather as a dye adsorbent

The reusability of BC-COF bio-leather as a dye adsorbent was evaluated by varying the concentration of the methylene blue aqueous solution, adsorption time, pH level of the aqueous solution, and the number of repeated adsorptions. For the evaluation, a methylene blue solution was used as the aqueous solution.

**Fig 8:**
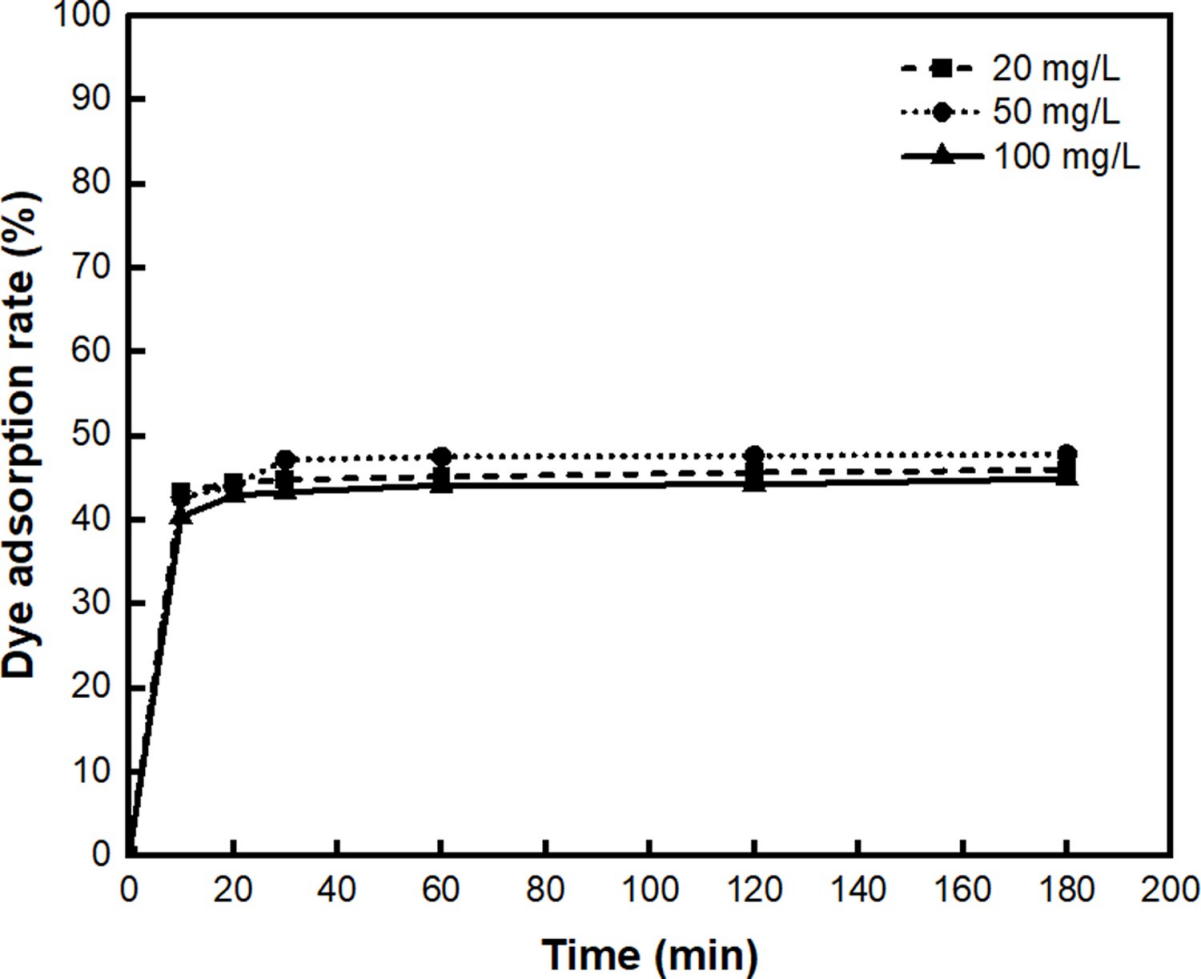
Dye adsorption rate (%) of BC-COF bio-leather with varied treatment time and concentration of the methylene blue solution (Extraction conditions: Liquor ratio of 1:10 (w/v), coffee at an amount of 200 wt% BC, 90°C, 1 h; dyeing conditions: Fabric-to-liquor ratio of 1:10 (w/v), 70°C, 30 min, meta-mordanting with 1% of FeSO_4_; dye adsorption conditions: Fabric-to-liquor ratio of 1:200 (w/v), 25°C).

First, the effective adsorption time and concentration of the methylene blue solution were tested (Fig 8). The dye adsorption of BC-COF bio-leather increased quickly within the initial 30 minutes and then reached equilibrium. In the initial stage of adsorption, there might be a large number of active sites on the surface of BC-COF that could react with methylene blue [66]. As the time elapsed for more than 30 minutes, the number of active sites decreased because of the repulsive forces between the adsorbed and free methylene blue molecules, attaining equilibrium [67]. In addition, the dye adsorption rate of BC-COF increased as the concentration of methylene blue solution increased, reaching its highest at 47.8% at a concentration of 50 mg/L. When the concentration of the methylene blue solution was increased to 100 mg/L, the dye adsorption rate slightly decreased to 43.3%. This could be due to the limited number of vacant active sites inside BC-COF that could adsorb methylene blue dye [66]. Thus, it was confirmed that BC-COF was the most effective in adsorbing the dye from 50 mg/L of methylene blue solution for 30 minutes. Moreover, although the dye adsorption rate was somewhat decreased as the concentration of methylene blue solution increased, BC-COF bio-leather showed that it could effectively adsorb the dye even at a high concentration of dye solution, such as 100 mg/L.

The dye adsorption rate of BC-COF bio-leather for methylene blue adsorption was compared with those of previously reported BC derived adsorbents. From Table 4, it was confirmed that BC-COF had relatively good dye adsorption rate compared to various adsorbents reported in the previous studies.

**Table 4:** Maximum dye adsorption rate (%) for various BC derived adsorbents for methylene blue dye adsorption.

Adsorbent	Dye adsorption rate (%)	Reference
BC-COF	47.8	This work
BC	53.0	Saleh et al., 2021 [68]
BC	27.9	Yi et al., 2021 [69]
BC	30.8	Almasi et al., 2020 [70]
Iron oxides@BC	53.8	Marsudi et al., 2021 [71]

**Fig 9:**
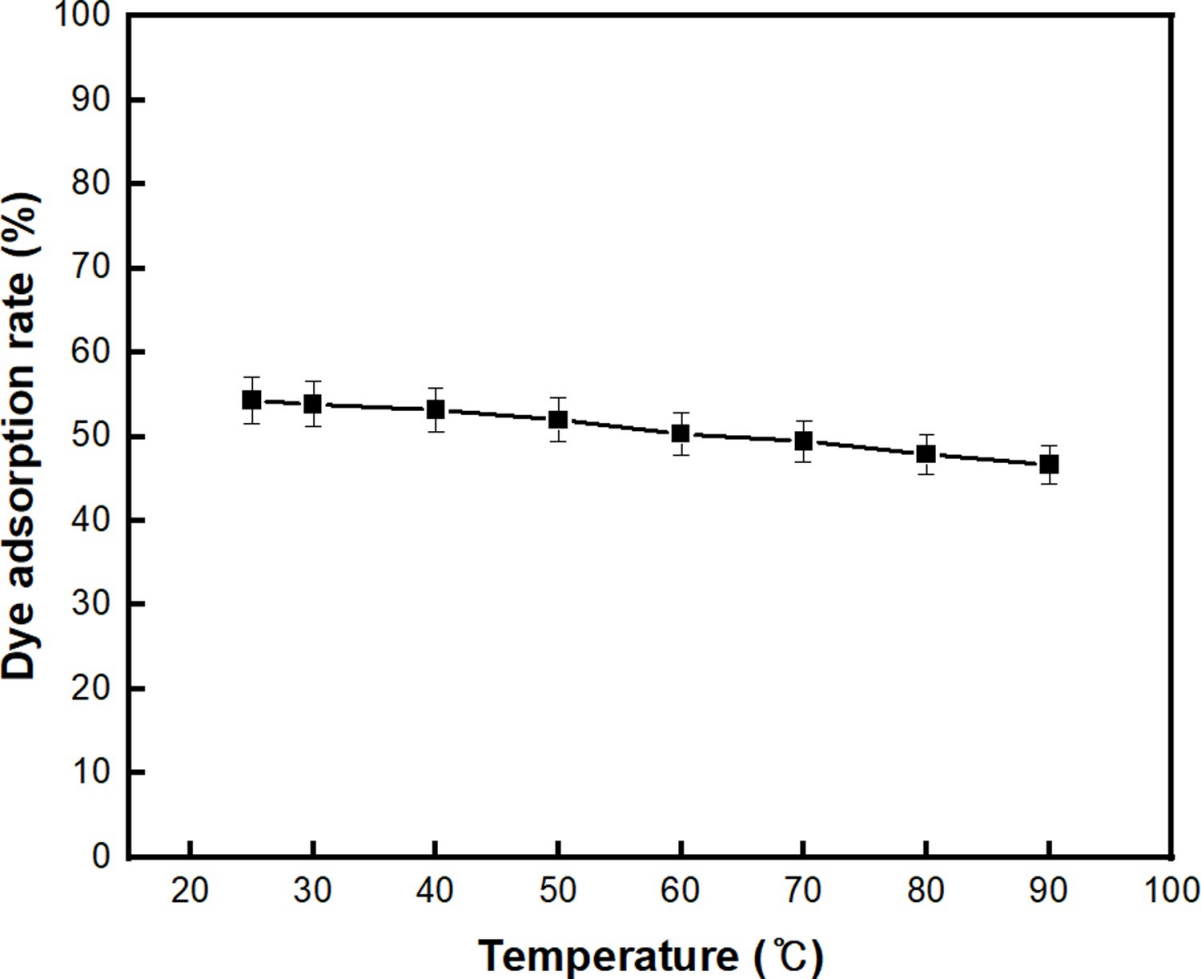
Dye adsorption rate (%) of BC-COF bio-leather with varied adsorption temperature (Extraction conditions: Liquor ratio of 1:10 (w/v), coffee at an amount of 200 wt% BC, 90°C, 1 h; dyeing conditions: Fabric-to-liquor ratio of 1:10 (w/v), 70°C, 30 min, meta-mordanting with 1% of FeSO_4_; dye adsorption conditions: 50 mg/L of methylene blue solution with a fabric-to-liquor ratio of 1:200 (w/v), 30 min).

Next, the effective adsorption temperature was analyzed. According to Fig 9, the dye adsorption rate of BC-COF reached the highest value of 54.3% at 25°C, and slightly decreased as the adsorption temperature increased. This result is similar to that of previous studies [36, 72]. It could be assumed that as the temperature increased, the adsorptive forces between methylene blue dyes and the functional groups on the surface of BC-COF were weakened [36]. Thus, it was confirmed that the temperature change had little effect on the adsorption of methylene blue using BC-COF.

**Fig 10:**
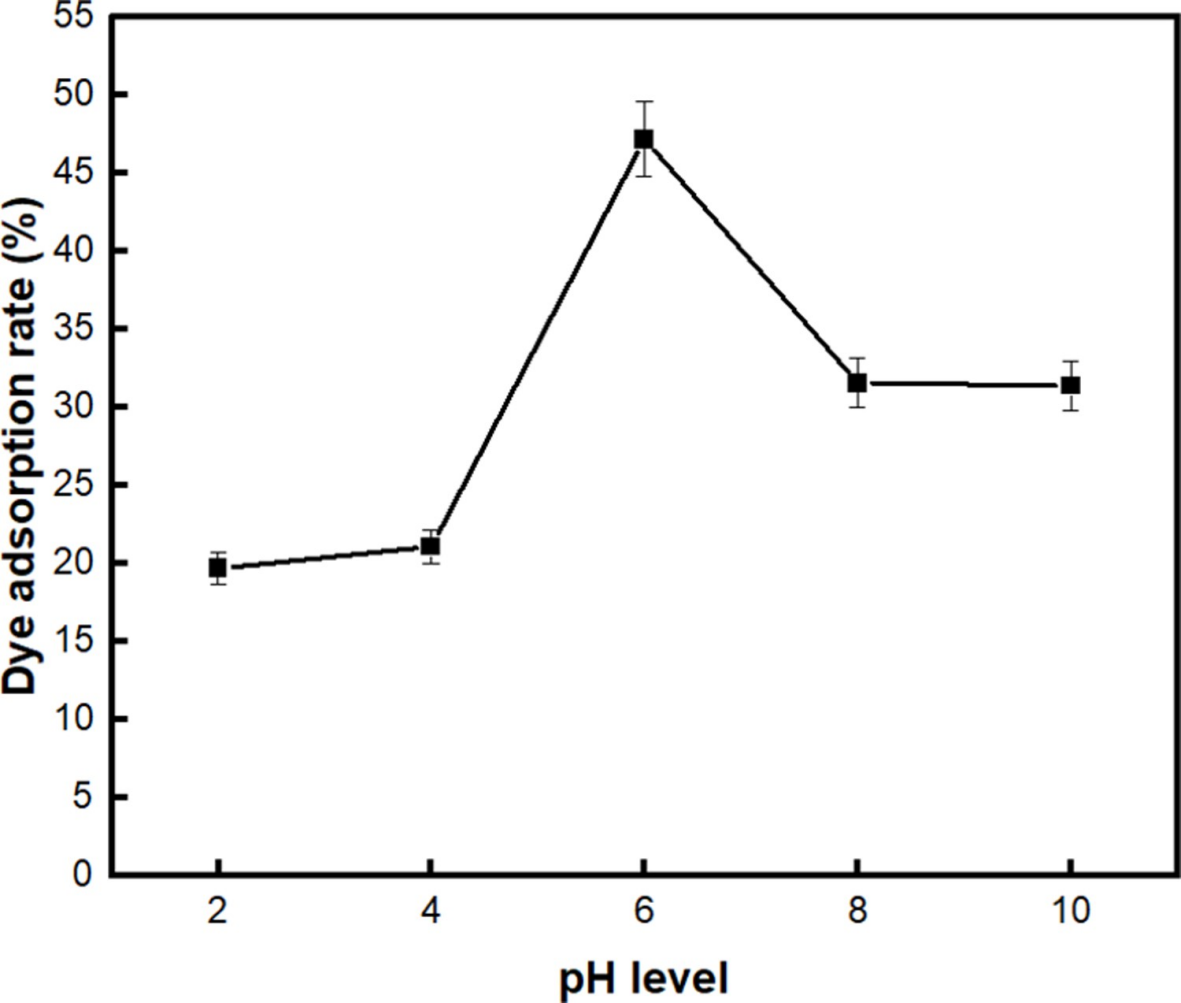
Dye adsorption rate (%) of BC-COF bio-leather with varied pH level of the methylene blue solution (Extraction conditions: Liquor ratio of 1:10 (w/v), coffee at an amount of 200 wt% BC, 90°C, 1 h; dyeing conditions: Fabric-to-liquor ratio of 1:10 (w/v), 70°C, 30 min, meta-mordanting with 1% of FeSO_4_; dye adsorption conditions: 50 mg/L of methylene blue solution with a fabric-to-liquor ratio of 1:200 (w/v), 25°C, 30 min).

Previous studies have found that the pH of the dye solution is an important factor influencing the adsorption of dyes [66, 73]. Thus, the effect of the pH level of the methylene blue solution on the adsorption properties of BC-COF bio-leather was evaluated. Fig 10 demonstrates that the dye adsorption rate of BC-COF increased as the pH level of the solution increased from pH 2 to 6, and reached the highest value of 47.2%. This could be explained by the fact that since methylene blue is a cationic dye, dye cations might compete with the positively charged H^+^ ions on the surface of BC-COF under acidic conditions [74]. As the pH level of the dye solution increased to pH 6, the surface the BC-COF became negatively charged and adsorbed the methylene blue cations by electrostatic attraction [75]. However, it was observed that the dye adsorption rate decreased when the pH level of the dye solution exceeded pH 6. This tendency might be explained that the mechanism for the methylene blue adsorption of BC-COF bio-leather is both the electrostatic interaction and the hydrogen bonding, which is consistent with a previous study of Yan and Wang [66].

The initial pH of the methylene blue solution affects the electric charge of the adsorbent and the electrostatic interaction between the adsorbent and the dye molecules, which in turn affects the methylene blue adsorption [76]. Thus, it is necessary to analyze the effect of the initial pH change of the dye solution on the adsorption rate.

**Fig 11:**
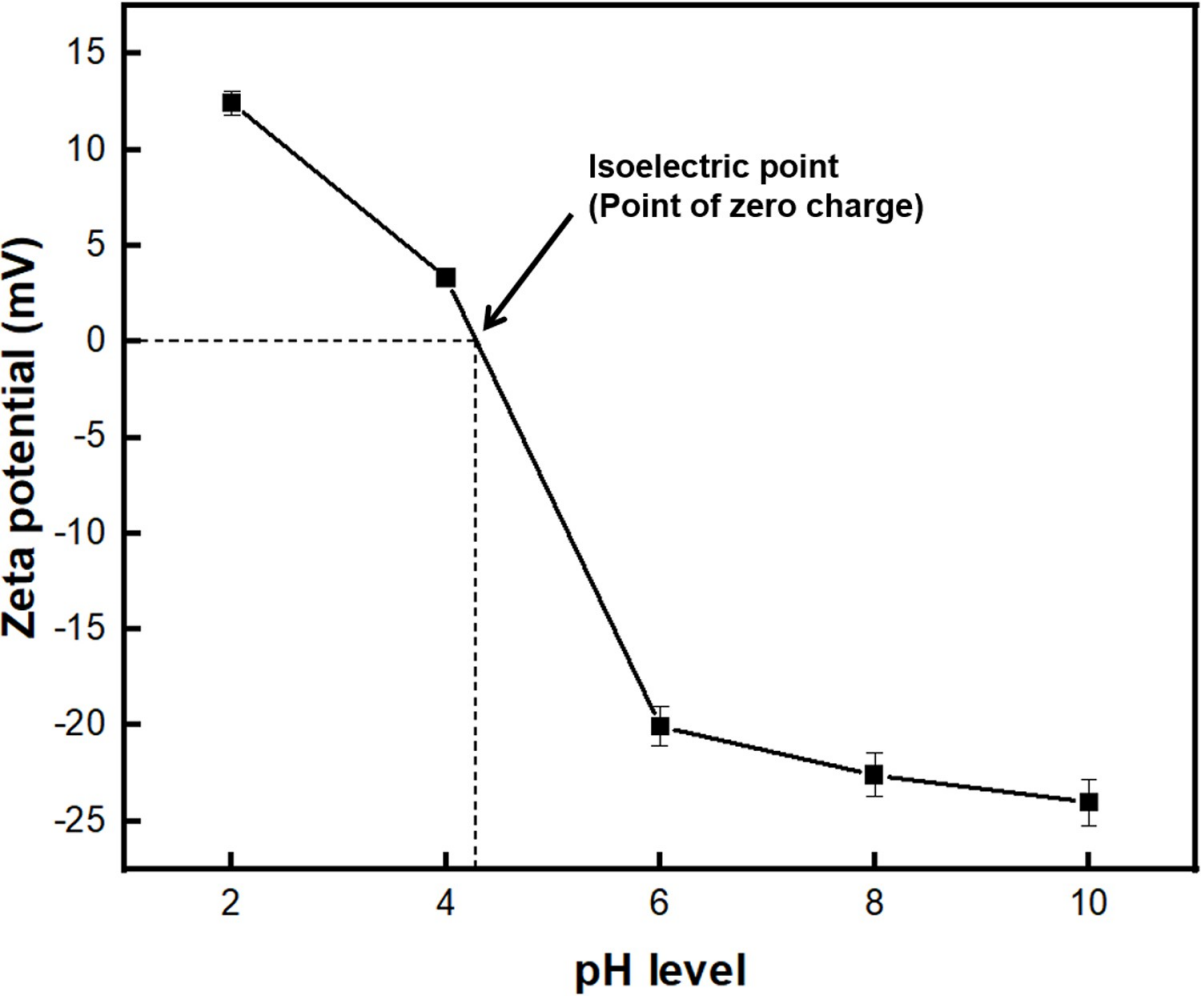
Zeta potential (mV) of BC-COF bio-leather with varied pH level (Extraction conditions: Liquor ratio of 1:10 (w/v), coffee at an amount of 200 wt% BC, 90°C, 1 h; dyeing conditions: Fabric-to-liquor ratio of 1:10 (w/v), 70°C, 30 min, meta-mordanting with 1% of FeSO_4_; dye adsorption conditions: 50 mg/L of methylene blue solution with a fabric-to-liquor ratio of 1:200 (w/v), 25°C, 30 min).

From the zeta potential analysis of BC-COF (Fig 11), the zeta potential value gradually decreased as the pH increased, and the isoelectric point was observed around pH 4. When pH level was lower than 4, the number of cations (H^+^ ions) increased and the hydroxyl functional groups on the surface of BC-COF were protonated [77]. As a result, BC-COF became positively charged and repulsed the cationic methylene blue dyes, thereby reducing the dye adsorption [78]. Meanwhile, when pH level was above 4, BC-COF was deprotonated and became negatively charged, methylene blue adsorption was improved by the strong electrostatic interaction formed between BC-COF and methylene blue molecules [79]. In addition, according to Fig 10, it was observed that some methylene blue molecules were adsorbed even at pH level below 4. This might be explained that methylene blue dyes are adsorbed by hydrogen bonding, π-π interaction from van der Waals force, etc. other than electrostatic interaction [77], as it was described above. Moreover, the absolute value of zeta potential of BC-COF did not significantly increase when pH level is higher than 6. This could be assumed that the zeta potential of BC-COF has reached the equilibrium state at pH 6, which was consistent with the result in Fig 10 that the highest methylene blue adsorption rate was observed at pH 6. Thus, the effective pH level of the methylene blue solution was found to be pH 6 for subsequent adsorption tests.

**Fig 12:**
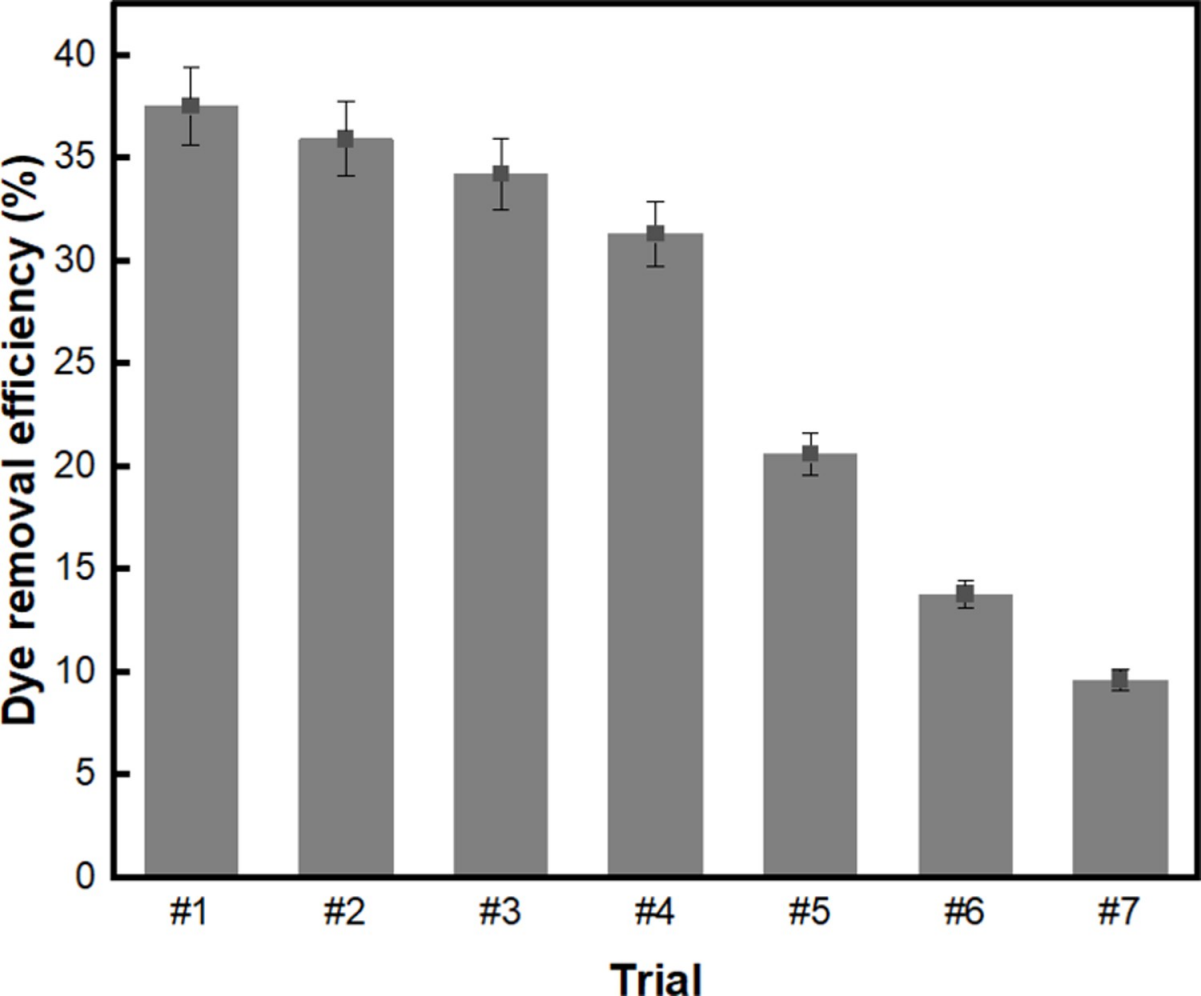
Dye adsorption rate (%) of BC-COF bio-leather with repeated dye adsorption trials (Extraction conditions: Liquor ratio of 1:10 (w/v), coffee at an amount of 200 wt% BC, 90°C, 1 h; dyeing conditions: Fabric-to-liquor ratio of 1:10 (w/v), 70°C, 30 min, meta-mordanting with 1% of FeSO_4_; dye adsorption conditions: 50 mg/L of methylene blue solution with a fabric-to-liquor ratio of 1:200 (w/v), pH 6, 25°C, 30 min).

Subsequently, repeated dye adsorption tests were conducted to examine the reusability of BC-COF bio-leather as a dye adsorbent. Fig 12 reveals that the BC-COF retained the dye adsorption rate of more than 30% after four repeated trials. The total decrease in the dye adsorption rate was approximately 6.2%, assuming that BC-COF has the potential to adsorb methylene blue from aqueous solution when it is reused four times. However, the dye adsorption rate of BC-COF decreased by approximately 10.7% after five repeated trials, and continuously decreased to 9.6% after seven repeated trials. Similar to the findings from a previous study, the decrease in the dye adsorption rate could be explained by the elimination of the coffee molecules inside the BC-COF [79]. Although the dye adsorption rate of BC-COF decreased when it was reused more than five times, the BC-COF continued to show a dye adsorption rate of 9.6% after seven repeated trials. Thus, it was confirmed that BC-COF bio-leather could be reused as a methylene blue adsorbent for multiple times.

**Fig 13:**
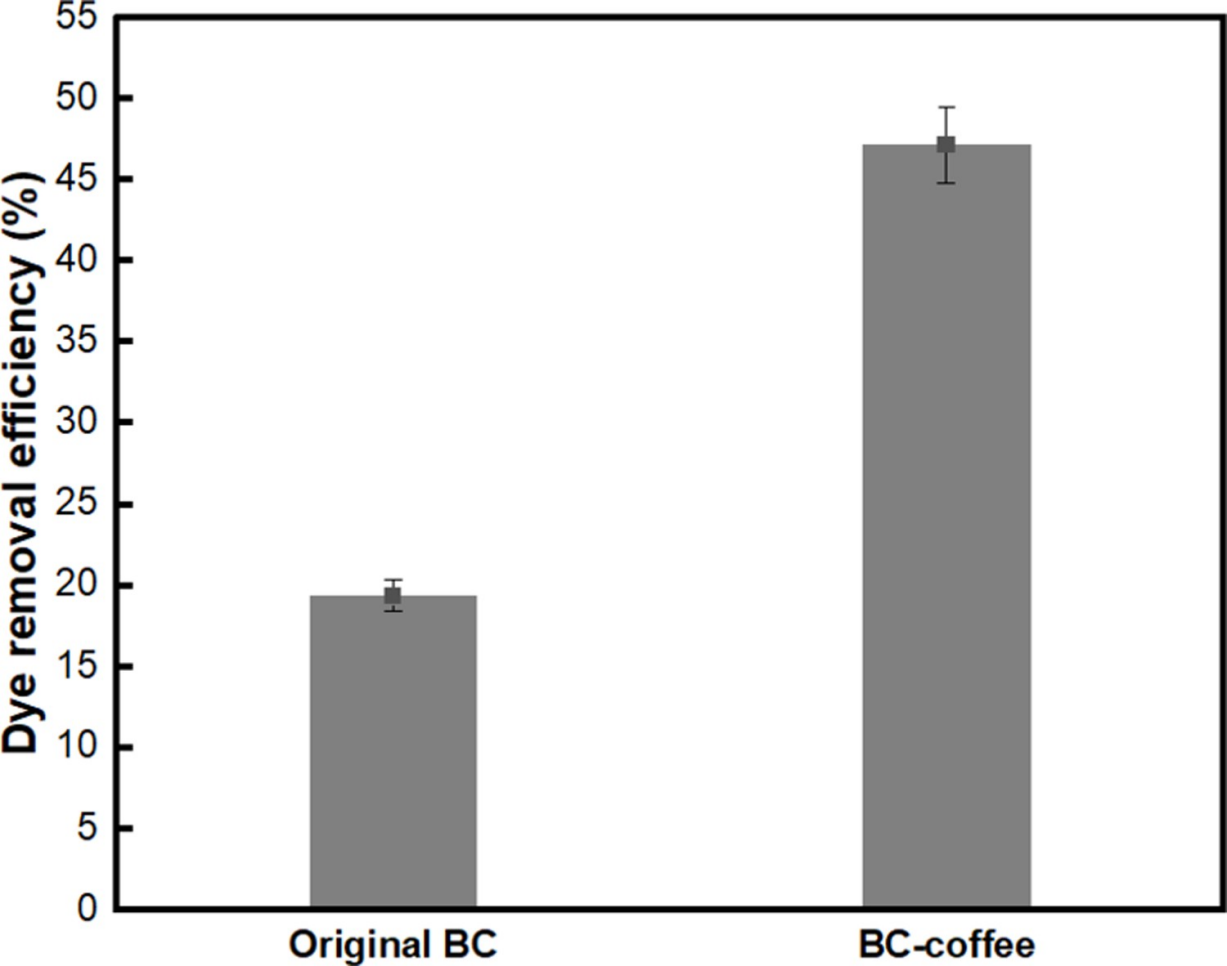
Dye adsorption rate (%) of original BC and BC-COF bio-leather (Extraction conditions: Liquor ratio of 1:10 (w/v), coffee at an amount of 200 wt% BC, 90°C, 1 h; dyeing conditions: Fabric-to-liquor ratio of 1:10 (w/v), 70°C, 30 min, meta-mordanting with 1% of FeSO_4_; dye adsorption conditions: 50 mg/L of methylene blue solution with a fabric-to-liquor ratio of 1:200 (w/v), pH 6, 25°C, 30 min).

To confirm the effect of coffee on the dye adsorption of BC-COF bio-leather, a comparative analysis with the original BC was conducted. As shown in Fig 13, the dye adsorption rate of BC-COF was 47.2%, whereas that of the original BC was 19.3%. Although the exact adsorption mechanism of coffee has not been reported yet, some studies have suggested that tannins, which are abundant in coffee, are effective in removing cationic dyes such as methylene blue [80]. Thus, it could be assumed that tannins inside BC-COF could effectively adsorb methylene blue. This might explain why BC-COF bio-leather has a better dye adsorption rate than the original BC.

## Conclusion

This study proposed a method for the natural dyeing of BC with coffee to develop an eco-friendly dyed BC bio-leather, and evaluated its reusability as a dye adsorbent. To select the dyeing condition with the highest color strength value, parameters such as the extraction of coffee, dyeing temperature, and dyeing time were examined. The dyeing condition of BC-COF bio-leather with the highest color strength value was found to be 70°C for 30 minutes with a fabric-to-liquor ratio of 1:10 (w/v). A washing durability test was conducted to determine the mordanting condition of BC-COF bio-leather with the lowest percent change in residual color strength. Consequently, meta-mordanted BC-COF bio-leather with 1% of FeSO_4_ retained about 49.4% of its original color after three repeated washing cycles. According to the FT-IR and XRD analyses, BC-COF bio-leather was successfully dyed with coffee, and the chemical and crystalline structures of BC were maintained after dyeing with coffee. Moreover, from FE-SEM and BET surface area analysis, it was confirmed that the brownish color on the BC-COF bio-leather was due to the penetrated coffee molecules inside the BC fiber structures. Subsequently, the reusability of BC-COF bio-leather as a dye adsorbent was evaluated using methylene blue dye. Parameters such as the concentration and pH level of methylene blue solution, adsorption time and temperature, and number of repeated adsorptions were determined by using UV-Vis spectroscopy and zeta potential measurement. BC-COF bio-leather was found to be most effective when pH 6 of 50 mg/L of methylene blue solution was adsorbed for 30 minutes at 25°C, and it could be reused as a methylene blue dye adsorbent for multiple times. Moreover, the dye adsorption rate of BC-COF bio-leather was about 2.4 times greater than that of the original BC. From the result, it was confirmed that coffee molecules inside BC-COF could improve the performance of BC-COF as a dye adsorbent. This study, however, has a limitation that metallic mordants were used to enhance the dyeability of BC-COF bio-leather. Thus, in further studies, it is necessary to avoid the use of metallic mordants to minimize the impact of heavy metals on the ecology.

## Supporting information

S1 File Supporting information for the manuscript. (DOCX)
