# Supplementary material for: Production of coffee-dyed bacterial cellulose as a bio-leather and using it as a dye adsorbent
Source: PLoS One. 2022 Mar 24;17(3):e0265743. doi: 10.1371/journal.pone.0265743 (PMC8947145; doi:10.1371/journal.pone.0265743)
Supplement: S1 File — (DOCX) [file pone.0265743.s001.docx]

**SI File. Supporting information for the manuscript.**

**SI Table 1.** The *K/S* values of BC-COF bio-leather at 440 nm with varied extraction liquor ratios used in Fig 1

| Fabric to liquor ratio (w/v) | *K/S* value |
| --- | --- |
| 1:10 | 15.82 ± 0.79 |
| 1:20 | 8.41 ± 0.42 |
| 1:50 | 4.11 ± 0.21 |
| 1:100 | 1.93 ± 0.10 |

**SI Table 2.** The *K/S* values of BC-COF bio-leather at 440 nm with varied dyeing temperature used in Fig 2

| Temperature (℃) | *K/S* value |
| --- | --- |
| 40 | 10.34 ± 0.52 |
| 50 | 10.68 ± 0.53 |
| 60 | 11.86 ± 0.59 |
| 70 | 15.82 ± 0.79 |
| 80 | 13.12 ± 0.66 |

**SI Table 3.** The *K/S* values of BC-COF bio-leather at 440 nm with varied dyeing time used in Fig 3

| Time | *K/S* value |
| --- | --- |
| 30 min | 15.82 ± 0.79 |
| 1 h | 14.38 ± 0.72 |
| 2 h | 13.23 ± 0.66 |
| 3 h | 11.87 ± 0.59 |
| 4 h | 11.56 ± 0.58 |
| 5 h | 11.41 ± 0.57 |

**SI Table 4.** The percent change in the residual *K/S* values of BC-COF bio-leather at 440 nm after three washing trials used in Fig 4

| Mordanting method | Mordant | Percent change in the residual *K/S* (%) | | |
| --- | --- | --- | --- | --- |
|  |  | Trial 1 | Trial 2 | Trial 3 |
| Non-mordanting | - | 88.37 | 39.29 | 12.70 |
| Pre-mordanting | AlK(SO_4_)_2_ | 50.66 | 30.43 | 29.72 |
|  | CuSO_4_ | 95.41 | 75.36 | 46.88 |
|  | FeSO_4_ | 89.81 | 40.97 | 40.97 |
| Meta-mordanting | AlK(SO_4_)_2_ | 90.1 | 34.57 | 16.14 |
|  | CuSO_4_ | 84.31 | 66.14 | 44.82 |
|  | FeSO_4_ | 93.42 | 78.59 | 49.43 |
| Post-mordanting | AlK(SO_4_)_2_ | 84.41 | 20.32 | 12.60 |
|  | CuSO_4_ | 91.91 | 44.40 | 25.50 |
|  | FeSO_4_ | 91.77 | 30.05 | 15.09 |

**SI Table 5.** Dye adsorption rate (%) of BC-COF bio-leather with varied treatment time and concentration of methylene blue solution used in Fig 8

| Time (min) | Dye adsorption rate (%) of varied concentrations of methylene blue solution | | |
| --- | --- | --- | --- |
|  | 20 mg/L | 50 mg/L | 100 mg/L |
| 0 | 0 | 0 | 0 |
| 10 | 43.35 | 42.65 | 40.39 |
| 20 | 44.40 | 44.06 | 42.92 |
| 30 | 44.82 | 47.18 | 43.32 |
| 60 | 45.17 | 47.53 | 44.15 |
| 120 | 45.66 | 47.65 | 44.22 |
| 180 | 45.98 | 47.82 | 44.94 |

**SI Table 6.** Dye adsorption rate (%) of BC-COF bio-leather with varied adsorption temperature used in Fig 9

| Temperature (℃) | Dye adsorption rate (%) |
| --- | --- |
| 25 | 54.31 ± 2.72 |
| 30 | 53.84 ± 2.69 |
| 40 | 53.16 ± 2.66 |
| 50 | 52.01 ± 2.60 |
| 60 | 50.30 ± 2.52 |
| 70 | 49.47 ± 2.47 |
| 80 | 47.92 ± 2.40 |
| 90 | 46.67 ± 2.33 |

**SI Table 7.** Dye adsorption rate (%) of BC-COF bio-leather with varied pH level of the methylene blue solution used in Fig 10

| pH level | Dye adsorption rate (%) |
| --- | --- |
| 2 | 19.7 ± 1.0 |
| 4 | 21.1 ± 1.1 |
| 6 | 47.2 ± 2.4 |
| 8 | 31.6 ± 1.6 |
| 10 | 31.4 ± 1.6 |

**SI Table 8.** Zeta potential (mV) of BC-COF bio-leather with varied pH level used in Fig 11

| pH level | Zeta potential (mV) |
| --- | --- |
| 2 | 12.45 ± 0.62 |
| 4 | 3.33 ± 0.17 |
| 6 | -20.07 ± 1.0 |
| 8 | -22.61 ± 1.13 |
| 10 | -24.02 ± 1.2 |

**SI Table 9.** Dye adsorption rate (%) of BC-COF bio-leather with repeated dye adsorption trials used in Fig 12

| Trials | Dye adsorption rate (%) |
| --- | --- |
| 1 | 37.55 ± 1.88 |
| 2 | 35.95 ± 1.80 |
| 3 | 34.26 ± 1.71 |
| 4 | 31.33 ± 1.57 |
| 5 | 20.62 ± 1.03 |
| 6 | 13.80 ± 0.69 |
| 7 | 9.61 ± 0.48 |

**SI Table 10.** Dye adsorption rate (%) of original BC and BC-COF bio-leather used in Fig 13

| Sample | Dye adsorption rate (%) |
| --- | --- |
| Original BC | 19.35 ± 0.97 |
| BC-COF | 47.20 ± 2.36 |
